# Regulation of the *ahpC* Gene Encoding Alkyl Hydroperoxide Reductase in *Mycobacterium smegmatis*

**DOI:** 10.1371/journal.pone.0111680

**Published:** 2014-11-03

**Authors:** Ha-Na Lee, Na-On Lee, Seung J. Han, In-Jeong Ko, Jeong-Il Oh

**Affiliations:** 1 Department of Microbiology, Pusan National University, Busan, Korea; 2 Department of Microbiology and Institute for Immunology and Immunological Diseases, Yonsei University, Seoul, Korea; 3 Korea Science Academy of KAIST, Busan, Korea; University of Padova, Medical School, Italy

## Abstract

The *ahpC* (*MSMEG_4891*) gene encodes alkyl hydroperoxide reductase C in *Mycobacterium smegmatis* mc^2^155 and its expression is induced under oxidative stress conditions. Two well-defined inverted repeat sequences (IR1 and IR2) were identified in the upstream region of *ahpC*. Using a *crp* (cAMP receptor protein: MSMEG_6189) mutant and *in vitro* DNA-binding assay, it was demonstrated that the IR1 sequence serves as a Crp-binding site and that Crp functions as an activator in the regulation of *ahpC* expression. The expression level of *ahpC* was shown to be proportional to intracellular cAMP levels. Intracellular levels of cAMP were increased in *M. smegmatis*, when it was treated with oxidative stress inducers. The IR2 sequence is very similar to the known consensus sequence of FurA-binding sites and involved in the negative regulation of *ahpC* expression. Taken together, these results suggest that the induction of *ahpC* expression under oxidative stress conditions probably results from a combinatory effect of both inactivation of FurA by oxidative stress and activation of Crp in response to increased levels of cAMP.

## Introduction

Alkyl hydroperoxide reductase C is a member of the peroxiredoxin family that reduce organic peroxides to their corresponding organic alcohols [1]. These enzymes from mycobacteria possess peroxinitrite reductase activity as well as peroxidase activity reducing both organic peroxides and hydrogen peroxide [2], [3], [4], [5]. Alkyl hydroperoxide reductase C is encoded by the *ahpC* gene in mycobacteria and contains two catalytically important cysteine residues, one of which (peroxidatic cysteine) is used to reduce the substrates (peroxides or peroxinitrite) with its concomitant oxidation to cysteine sulfenic acid. The sulfhydryl group of the other cysteine residue (resolving cysteine) attacks the peroxidatic cysteine sulfenic acid to form a disulfide bond [6], [7]. The mycobacterial AhpC forms a homodimer as a minimum functional unit in which the resolving cysteine from one subunit acts on the peroxidatic cysteine in the other subunit [2], [8]. X-ray diffraction analysis of crystallized AhpC revealed that AhpC has the structure of a ring-shaped hexamer of dimers [7]. The disulfide bond formed between the peroxidatic and resolving cysteine residues in AhpC is reduced for the next catalytic cycle by the AhpD peroxiredoxin reductase [2], [6], [7]. The reducing equivalents for the reduction of the oxidized AhpC are transferred to AhpD from NADH via dihydrolipoamide succinyltransferase (SucB) and dihydrolipoamide dehydrogenase (Lpd) [9]. It was reported that AhpC can be also reduced by thioredoxin C (TrxC) and NADPH-dependent thioredoxin reductase [10]. The *ahpC* gene forms an operon with its downstream gene, *ahpD* [11]. Genes encoding the OxyR homologs, which are LysR family regulators and involved in peroxide stress response, are divergently located upstream of the *ahpCD* operons in most mycobacteria [12]. However, the *oxyR* genes identified in *Mycobacterium tuberculosis* and other members of the *M. tuberculosis* complex (*Mycobacterium bovis*, *Mycobacterium africanum*, and *Mycobacterium microti*) are inactivated by numerous mutations and *Mycobacterium smegmatis* does not have the *oxyR* gene [12], [13]. Despite the lack of the functional *oxyR* genes, expression of *ahpC* was reported to be induced in *M. bovis* BCG in the presence of diamide and synthesis of AhpC in *M. smegmatis* was shown to be inducible by both hydrogen peroxide and organic hydroperoxides such as cumene hydroperoxide (CHP) and *tert*-butyl hydroperoxide (BHP) [14], [15], indicating that these bacteria possess other regulatory system(s) responding to oxidative stress and regulating *ahpC* expression. An *ahpC* mutant of *M. tuberculosis* is more susceptible to CHP than the wild type [16]. It was also demonstrated that *ahpC* expression was derepressed in a virulent *M. tuberculosis* strain grown under static growth conditions, suggesting the possibility that depletion of oxygen might lead to derepression of *ahpC* [16]. Overexpression of the *oxyS* gene was demonstrated to reduce the level of AhpC in *M. tuberculosis*, and microarray analyses revealed that expression of *ahpC* was downregulated in Crp (cAMP receptor protein; Rv3676) and SenX3-RegX3 two-component system mutants and upregulated in a WhiB4 mutant, compared with the wild-type strain of *M. tuberculosis* [17], [18], [19], [20]. These findings indicate the possible involvement of OxyS, Crp, WhiB4, and SenX3-RegX3 TCS in the regulation of *ahpC* expression. It was also reported that expression of *ahpC* was not changed in a SigF mutant of *M. smegmatis*, ruling out the involvement of SigF in the regulation of *ahpC* expression [21]. Despite a number of reports regarding *ahpC* expression, detailed regulatory mechanisms by which expression of *ahpC* is regulated in response to oxidative stress still remains elusive.

The Crp protein is a transcriptional regulator that responds to intracellular fluctuation of the cAMP level [22]. *M. tuberculosis* Crp (Crp_Mtb_) consists of the N-terminal cAMP-binding domain (residues 1–114) and the C-terminal DNA-binding domain (residues 146–233) that are connected by a hinge region (residues 117–144) [23]. Three-dimensional structures of the cAMP-bound and cAMP-free Crp_Mtb_ revealed that Crp_Mtb_ forms homodimer like *Escherichia coli* Crp and it undergoes allosteric conformational changes by cAMP binding [24], [25]. The binding affinity of Crp_Mtb_ for cAMP is lower than that of *E. coli* Crp and cAMP binding to Crp_Mtb_ is not cooperative [26], [27]. These properties were suggested to render Crp_Mtb_ responsive to changes in the cAMP level in the background of high cAMP concentrations within mycobacterial cells [27]. Conformational changes of Crp_Mtb_ by cAMP binding were proposed to lead to a small increase (∼2 fold) in its binding affinity for the target DNA sequence (TGTGA-N_6_-TCACA) [26]. Growth of *M. tuberculosis* was shown to be compromised in both macrophages and a mouse infection model by the inactivation of the *crp* gene [18]. Internalization of mycobacteria into macrophages resulted in a surge in cAMP production by mycobacteria [28], [29]. Both findings imply that some genes involved in the enhanced survival of mycobacteria in hostile environments such as oxidative and nitrosative stress conditions within macrophages might be regulated by Crp.

The FurA proteins in mycobacteria are members of the ferric uptake regulator (Fur) family. FurA is composed of the N-terminal domain containing a helix-turn-helix motif for DNA binding and the C-terminal metal-binding domain. The Fe^2+^-binding form of FurA functions as an active transcriptional regulator that control expression of genes involved in iron homeostasis and oxidative stress response [30], [31] In mycobacteria there are also FurB proteins belonging to the Fur family. FurB is a Zn^2+^-dependent regulator that regulates genes related to zinc homeostasis [32].

Here, we report that expression of the *ahpC* gene, whose product is implicated in detoxification of peroxides and peroxinitrite, is regulated by Crp and probably FurA in *M. smegmatis*.

## Materials and Methods

### Strains, plasmids, and growth conditions

The bacterial strains and plasmids used in this study are listed in Table 1. *E. coli* strains were grown in Luria-Bertani (LB) medium at 37°C as described elsewhere [33]. *M. smegmatis* strains were grown aerobically at 37°C in Middlebrook 7H9 medium (Difco, Sparks, MD) supplemented with 0.2% (wt/vol) glucose as a carbon source and 0.02% (vol/vol) Tween 80 as an anticlumping agent. For iron-depleting growth conditions of mycobacterial cultures, MOPS-defined medium was used in place of 7H9 medium. The MOPS medium is composed of 25 mM MOPS (pH 7.2), 25 mM KCl, 10 mM Na_2_SO_4_, 20 mM NH_4_Cl, 10 mM K_2_HPO_4_, 2 mM MgSO_4_, and 0.1 mM CaCl_2_. When antibiotics were required, ampicillin (100 µg/ml for *E. coli*), kanamycin (50 µg/ml for *E. coli* and 15 µg/ml for *M. smegmatis*) and hygromycin (200 µg/ml for *E. coli* and 50 µg/ml for *M. smegmatis*) were added to the medium. For treatment of *M. smegmatis* cultures with various oxidative and nitrosative stress conditions, *M. smegmatis* strains were grown until an optical density at 600 nm (OD_600_) reached 0.4 to 0.5 on a gyratory shaker (200 rpm). Following the addition of stress-inducing reagents to the cultures, the strains were further grown for 1 h. The working concentrations of the reagents are as follows: 100 µM cumene hydroperoxide (CHP), 100 µM plumbagin (PB), 5 mM diamide, 15 mM hydrogen peroxide (H_2_O_2_), 10 mM sodium ascorbate (VC), and 10 mM sodium nitroprusside (SNP).

**Table 1:** Bacterial strains and plasmids used in this study.

Strain or plasmid	Relevant phenotype or genotype	Reference or source
**Strains**		
*E. coli* DH5α	φ80d*lacZ*ΔM15 Δ*lacU169 recA1 endA1 hsdR17 supE44 thi1 gyrA96 relA1*	[59]
*E. coli* HB101	F *supE44 ara14 galK2* _(*gpt-proA*)*62 lacY1 hsdS20 rpsL20 xyl-5 mtl-1 recA13* _(*mcrC-mrr*)	Stratagene
*E. coli* BL21 (DE3)	F^−^ *omp*T *hsd S*_B_(r_B_^−^m_B_^−^) *dcm gal*λ (DE3)	Promega
*M. smegmatis* mc^2^155	High-transformation-efficiency mutant of *M. smegmatis* ATCC 607	
*M. smegmatis crp*	*crp* (*MSMEG_6189*) insertion mutant derived from *M. smegmatis* mc^2^155	This study
**Plasmids**		
pGEM-T Easy	Amp^r^; linear plasmid derived from pGEM5-Zf	Promega
pMH702	Hyg^r^; a derivative of pYUB572, its ampicillin-resistance gene is replaced by ahygromycin-resistance gene and the gene is flanked by two 34-bp *loxP* sequences	Yang JY, unpublished
phAE159	Deletion mutant of mycobacteriophage TM4	[60]
pKOTs	Hyg^r^; pKO-based vector containing a temperature-sensitive replication origin (pAL500Ts) and pUC ori	[61]
pMV306	Km^r^; integrative vector containing the *int* and *attP* sites of mycobacteriophageL5 for integration into the mycobacterial genome	[62], [63]
pBluescript II KS +	Amp^r^; *lacPOZ*’	Stratagene
pNC	Hyg^r^; promoterless *lacZ*	[64]
pProEX HTa	Amp^r^; Trc promoter, instrinsic 6 His tag	Invitrogen
pMH201	Km^r^; acetamide-inducible promoter, derivative of pMV306	[65]
pProcrpHis	pProEX HTa with 681-bp EcoRI-XhoI fragment containing *crp* of *M. smegmatis* mc^2^155 with 6 His codons	This study
pMV306crp	pMV306 with 1,004-bp NotI-HindIII fragment containing *crp* of *M. smegmatis* mc^2^155	This study
pBSahpC	pBluescript II KS+with 836-bp XbaI-ClaI fragment containing the *ahpC* promoter region	This study
pBSM1	pBSahpC in which the nucleotide G within the Crp-binding site is substituted with C	This study
pBSM2	pBSahpC in which the nucleotide C within the Crp-binding site is substituted with G	This study
pBSM3	pBSahpC derivative that containing the 12-bp-deleted IR2 sequence in the *ahpC* control region	This study
pNCahpC	pNC with 836-bp XbaI-ClaI fragment from pBSahpC	This study
pNCM1	pNC with 836-bp XbaI-ClaI fragment from pBSM1	This study
pNCM2	pNC with 836-bp XbaI-ClaI fragment from pBSM2	This study
pNCM3	pNC with 830-bp XbaI-ClaI fragment from pBSM3	This study
pMHpdeHis	pMH201 with 1,011–bp NdeI-XbaI fragment containing the *rv0805* gene of*M. tuberculosis* with 6 His codons before its stop codon	This study

### DNA manipulation and electroporation

Standard protocols and manufacturers’ instructions were followed for recombinant DNA manipulations. The transformation of *M. smegmatis* with plasmids was carried out by electroporation as described elsewhere [34].

### Construction of plasmids

(i) pProcrpHis: A 681-bp DNA fragment including the *crp* (*MSMEG_6189*) gene was amplified with F_crp (5′- GAATTCATGGACGAGATCCTGGCCAG-3′) and R_crp (5′- CTCGAGCTAGCGGGCGCGGCGGGCCA-3′) using *M. smegmatis* genomic DNA as a template and *Pfu* DNA polymerase. The PCR product was restricted with EcoRI and XhoI and inserted into pProEX HTa digested with the same enzymes, yielding pProcrpHis that was used to overexpress the N-terminally His_6_-tagged Crp protein. (ii) pMV306crp: pMV306crp was constructed for complementation of the *crp* mutant with the intact *crp* gene. A 1,004-bp DNA sequence containing the *crp* gene was amplified with the primer set, F_pMV306crp (5′-AATTGCGGCCGCCCCGCGAGCAGGCACCAC-3′) and R_pMV306crp (5′-CCGGAAGCTTTTCGGCGAACGGGGCGAG-3′) using *M. smegmatis* genomic DNA as a template. The DNA fragment obtained from the PCR reaction was digested with NotI and HindIII and ligated with pMV306 restricted with the same restriction enzymes, resulting in pMV306crp. (iii) pNCahpC, pNCM1, pNCM2, and pNCM3: pNCahpC, pNCM1, pNCM2, and pNCM3 are *ahpC*::*lacZ* transcriptional fusion plasmids. For the construction of pNCahpC, a 836-bp DNA fragment comprising the 5′ portion (99 bp) of *ahpC* and the 737-bp DNA sequence upstream of *ahpC* was amplified with the primers, F_ahpC (5′-AAAATCTAGACATCGACGTCGCCCGCCC-3′) and R_ahpC (5′-GACAATCGATGTCATCGGGCTGCTTGG-3′). The PCR product was digested with ClaI and XbaI and cloned into pBluescript II KS+, resulting in pBSahpC. pBSahpC was restricted with ClaI and XbaI and the 836-bp fragment was cloned into pNC, yielding the pNCahpC. To construct pNCM1 and pNCM2, PCR-based site-directed mutagenesis was carried out using pBSahpC as a template. Synthetic oligonucleotides 33 bases long and containing substituted nucleotides in the middle of their sequences were used to mutagenize the Crp-binding site (IR1). Mutations were verified by DNA sequencing. The 836-bp XbaI and ClaI fragments from the mutated pBSM1 and pBSM2 were cloned into pNC, resulting in the plasmids pNCM1 and pNCM2, respectively. In order to construct pNCM3, inverse PCR was conducted with the primers, L_M3 (5′-ATATGGATCCCCAGATTTACACCACGATTCTGGTC-3′, the BamHI site is underlined) and R_M3 (5′-ATATGGATCCAAACAAGAACACGTAGATGGGATGC-3′), and pBSahpC as a template to amplify the linear pBSahpC with BamHI sites at both ends. The amplified products were restricted with BamHI and self-ligated, resulting in the plasmid pBSM3 containing a BamHI site in place of IR2. pNCM3 was constructed by cloning the ClaI-XbaI DNA fragment from pBSM3 into pNC. (iv) pMHpdeHis: To construct pMHpdeHis for expression of cyclic nucleotide phosphodiesterase (Rv0805) of *M. tuberculosis*, PCR reaction was performed with the primers, F_rv0805 (5′-ATATCATATGCAATGGAGAGGGTTGGCACCTCAG-3′) and R_rv0805 (5′-ATATTCTAGATCAGTGATGGTGATGGTGATGGTCGACGGGACTTCGCGG-3′) and *M. tuberculosis* H37Rv genomic DNA as a template. The PCR product containing the *rv0805* gene with 6 His codons immediately before its stop codon was digested with NdeI and XbaI and cloned into pMH201, yielding pMHpdeHis.

### Construction of a *crp* mutant

A *crp* mutant in which the *crp* (*MSMEG_6189*) gene is disrupted by the insertion of a hygromycin-resistance gene, was constructed by one-step homologous recombination using the conditionally replicating shuttle phasmid vector phAE159 as previously described [35], [36]. Briefly, a 970-bp DNA fragment containing the 5′ portion (60 bp) of *crp* flanked with the 910-bp *crp* upstream sequence and a 963-bp DNA fragment containing the 3′ portion (60 bp) of *crp* flanked with the 903-bp *crp* downstream sequence (left and right arms, respectively) were amplified by PCR using *M. smegmatis* genomic DNA as a template with the primer sets, CrpL_F_BglII (5′-AGATCTGTCGAAGCGCTCGACGAGTTCCTGG-3′) and CrpL_R_SpeI (5′-ACTAGTCGCAACGGCGGTGGGTTCGA-3′) for the left arm and CrpR_F_NcoI (5′- CCATGGCTGGAGGGCAAGAGCGTGCT-3′) and CrpR_R_NcoI (5′-CCATGGCGTCGAGGTCGAGATCATCG-3′) for the right arm. Both PCR products were cloned into pGEM-T (Promega, Madison, WI), resulting in pGcrpL and pGcrpR. The plasmid pGcrpL was restricted with BglII and SpeI, and pGcrpR with NcoI. The DNA fragments were cloned into the cosmid pMH702 to flank the hygromycin-resistance gene cassette on both sides. The resulting cosmid pMH702crp was linearized with PacI and ligated with the PacI-digested shuttle phasmid phAE159. The ligation mixture was packaged using MaxPlax lambda packaging extracts (Epicentre Biotechnologies, Madison, WI) and transfection of *E. coli* HB101 was performed. Recombinant phasmids were isolated from hygromycin-resistant clones of *E. coli*. Transformation of *M. smegmatis* with the isolated phasmid at 30°C resulted in the generation of recombinant TM4 phages carrying the recombinant phasmid. The *crp* mutant was selected on hygromycin-containing 7H9 plates at 37°C following transfection of *M. smegmatis* with the recombinant TM4 phages. The mutation was confirmed by PCR.

### Reverse-transcription PCR (RT-PCR) and quantitative real-time PCR (qRT-PCR)

RNA isolation from *M. smegmatis* strains, preparation of cDNA, RT-PCR, and qRT-PCR were performed as described elsewhere [37]. The primers used in RT-PCR and qRT-PCR were listed in Table 2.

**Table 2:** The primers used for RT-PCR and qRT-PCR in this study.

Primer	Sequence (5′ to 3′)
16S rRNA_Forward	CTGGGACTGAGATACGGC
16S rRNA_Reverse	ACAACGCTCGGACCCTAC
ahpC_Forward	GTGTGTCGGTGGACA ACGAG
ahpC_Reverse	GGTCACCGACACGAACTGGA
pNClacZ_Forward	GGCGTTACCCAACTTAATCG
pNClacZ_Reverse	ACGACGACAGTATCGGCCTC

### Purification of Crp protein

N-terminally His_6_-tagged Crp protein was overexpressed in the *E. coli* BL21 (DE3) strain harboring pProcrpHis. The strain was grown aerobically at 37°C in LB medium containing 100 µg/ml ampicillin to an OD_600_ of 0.4 to 0.6. Expression of the *crp* gene was induced by the addition of isopropyl-β-D-thiogalactopyranoside (IPTG) to a final concentration of 0.5 mM, and cells were further grown at 30°C for 4 h. Harvested cells from 300 ml culture were resuspended in 5 ml buffer A [20 mM Tris-HCl (pH 8.0) and 100 mM NaCl] and disrupted by two passages through a French pressure cell. Following DNase Ι treatment (10 units/ml) in the presence of 10 mM MgCl_2_ for 30 min on ice, cell-free crude extracts were obtained by centrifugation twice at 20,000 × *g* for 10 min. 0.5 ml of the 80% (vol/vol) slurry of Ni-Sepharose high-performance resin (GE Healthcare, Piscataway, NJ) was added to the crude extracts and mixed gently by shaking for 2 h on ice. The protein-resin mixture was packed into a column. The resin was washed with 40 bed volumes of buffer A containing 5 mM imidazole, 40 bed volumes of buffer A containing 10 mM imidazole, 20 bed volumes of buffer A containing 50 mM imidazole, and then His_6_-tagged Crp was finally eluted with 13 bed volumes of buffer A containing 200 mM imidazole. Fractions from the elution step were collected and desalted by means of a PD-10 desalting column (GE Healthcare) equilibrated with 20 mM Tris-HCl (pH 8.0).

### Determination of the protein concentration

The protein concentration was determined by using the Bio-Rad protein assay kit (Bio-Rad, Hercules, CA) with bovine serum albumin as a standard protein.

### Western blotting analysis

To determine the amount of AhpC protein in cells, Western blotting analysis was performed as described elsewhere [38]. Rabbit polyclonal antibodies against AhpC were used at a 1∶2,000 dilution. Alkaline phosphatase-conjugated anti-rabbit IgG (Sigma, St. Louis, MO) was used at a 1∶5,000 dilution for the detection of the primary antibody.

### Zone inhibition assay

*M. smegmatis* strains were cultivated in 7H9 medium aerobically until OD_600_ reached 0.45. 5 ml of cultures were poured onto 7H9 plates. The plate surfaces were spread uniformly with the cultures and then the rest of the cultures were drained off. The plates were tapped on a paper towel to remove the remaining culture liquid. The plates were dried at room temperature for 3 to 4 h. The paper discs soaked with 15 µl of 1 and 2% (wt/vol) of CHP were placed onto the dried plates. The plates were incubated at 37°C for 3 days to observe zones of growth inhibition.

### Electrophoretic mobility shift assay (EMSA)

EMSA was carried out by using the Electrophoretic Mobility Shift Assay (EMSA) kit (Invitrogen, Carlsbad, NJ) according to the manufacturer’s instruction. 150-bp DNA fragments containing the wild-type or mutated IR1 sites were used in the assay. The DNA fragments were generated by PCR using the primer set, EMSA_150_F (5′-TCTGGTCGCGCCCTCTTAC-3′) and EMSA_150_R (5′-GGCAGACCGCATCCGCGG-3′) and pBSahpC, pBSM1, and pBSM2 as templates to obtain the corresponding DNA fragments. Reaction mixtures for DNA-protein binding were composed of appropriate amounts of DNA (2 µl), purified Crp (5 µl), distilled H_2_O (1 µ), and 5× binding buffer included in the kit (2 µl). cAMP was added to a final concentration of 100 µM. The binding reaction mixtures were incubated for 20 min at room temperature. After the addition of 2 µl of 6× loading buffer (included in the kit), the mixtures were subject to nondenaturing PAGE [8% (wt/vol) acrylamide] in 0.5× TBE buffer (41.5 mM Tris-borate and 0.5 mM EDTA, pH 8.3) at 14 V/cm for 2 h 20 min at 4°C. The gels were stained with the SYBR green staining solution (Invitrogen).

### β-galactosidase assay

The β-galactosidase activity was measured spectrophotometrically as described previously [39].

### Determination of the intracellular cAMP concentration

*M. smegmatis* cells corresponding to 1 ml of cultures at OD_600_ of 0.4 were harvested. Cell pellets were resuspended in 1 ml of 0.1 M HCl and then incubated for 10 min. Cells were disrupted once by using a Fastprep 120 beadbeater (Thermo, Milford, MA) at 6.5 m/sec for 45 sec. Cell-free supernatants were obtained by centrifugation at 13,400×*g* for 10 min. Using the prepared supernatants, the concentration of intracellular cAMP was determined by using the DetectX Direct Cyclic AMP Enzyme Immunoassay kit (Arbor Assays, Ann Arbor, MI) and a Microplate Reader (Bio-Rad) following the manufacturers’ instructions.

## Results

### Identification of *cis*-acting elements in the upstream region of *ahpC*

**Figure 1:**
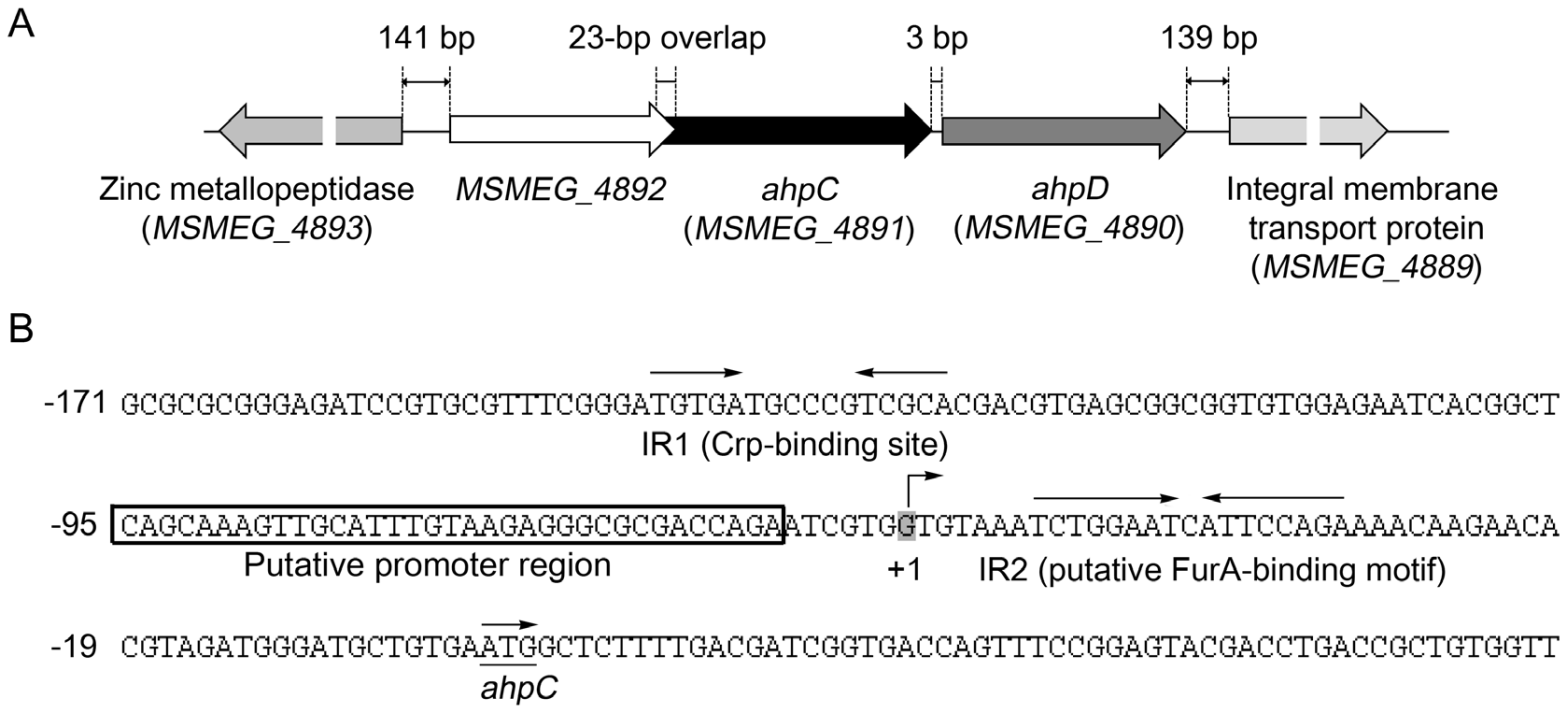
Genetic organization of the *ahpC* locus in *M. smegmatis* mc^2^155 (A) and the upstream sequence of *ahpC* encompassing its promoter region and the putative *cis*-acting elements involved in the regulation of *ahpC* expression (B). The lengths of the overlapping region between *MSMEG_4892* and *ahpC* and the intergenic regions are given as the nucleotide numbers above the schematic diagram. The two inverted repeats, IR1 (Crp-binding site) and IR2 (putative FurA-binding site), are marked by the two head-facing arrows above their sequences. The putative promoter region of *ahpC* is boxed. The nucleotide reported to be the transcriptional start point (+1) of *ahpC* is shaded in gray [15]. The start codon of *ahpC* is underlined and the arrow above it indicates the transcriptional direction. The numbers on the left of the sequences indicate the positions of the leftmost nucleotides relative to the *ahpC* gene.

The *ahpC* (*MSMEG_4891*) gene forms an operon together with *ahpD* (*MSMEG_4890*) in *M. smegmatis* [11]. The *MSMEG_4892* gene annotated as a hypothetical protein gene is located upstream of *ahpC* with a 23-bp overlap (Figure 1A). *MSMEG_4892* differs in its codon preference from other genes of *M. smegmatis* and its deduced protein product has no obvious similarity to other known proteins. When RT-PCR was performed with the primers that can detect mRNA encompassing both *MSMEG_4892* and *ahpC*, no PCR product was obtained (data not shown), indicating that *MSMEG_4892* does not form the same transcriptional unit with *ahpCD*.

The DNA sequence upstream of *ahpC* was analyzed to identify *cis*-acting elements involved in expression of *ahpC* (Figure 1B). The transcriptional start point of *ahpC* was reported previously [15]. The promoter region of *ahpC* around 10 and 35 bp upstream of the transcriptional start point does not have sequences similar to the consensus sequence of the mycobacterial −10 and −35 regions for the SigA sigma factor, indicating that *ahpC* has either a weak promoter or a promoter recognized by alternative sigma factors [40], [41]. As an initial attempt to identify regulatory systems that are responsible for the regulation of *ahp*C expression, we searched for inverted repeat sequences in the upstream sequence of *ahpC* under the presumption that multimeric regulatory proteins normally bind to their target DNA sequences with a dyad symmetry. Two well-defined inverted repeat sequences (IR1 and IR2) were identified and one of them (IR1) almost perfectly matched the consensus sequence of *E. coli*’s Crp-binding sites (TGTGA-N_6_-TCACA) [42]. A perfect inverted repeat sequence (IR2: TCTGGAAT-C-ATTCCAGA) was identified immediately downstream of the transcriptional start point. The IR2 sequence is highly similar to the sequence of a “FurA box” serving as the FurA-binding sequence in mycobacteria [43].

### Positive regulation of *ahpC* by Crp

There are two genes encoding Crp homologs (MSMEG_6189 and MSMEG_0539) in *M. smegmatis* genome. Since MSMEG_6189 showed a higher identity (98%) to *M. tuberculosis* Crp than MSMEG_0539 (78%), a *crp* (*MSMEG_6189*) mutant of *M. smegmatis* was first constructed to examine whether Crp is involved in the regulation of *ahpC* expression. Growth of the mutant was slower and reached the stationary phase at a lower cell density than that of the wild type, when both strains were grown aerobically in 7H9 medium supplemented with glucose (the doubling times of the wild type and the mutant were 4.2 and 4.9 h, respectively).

**Figure 2:**
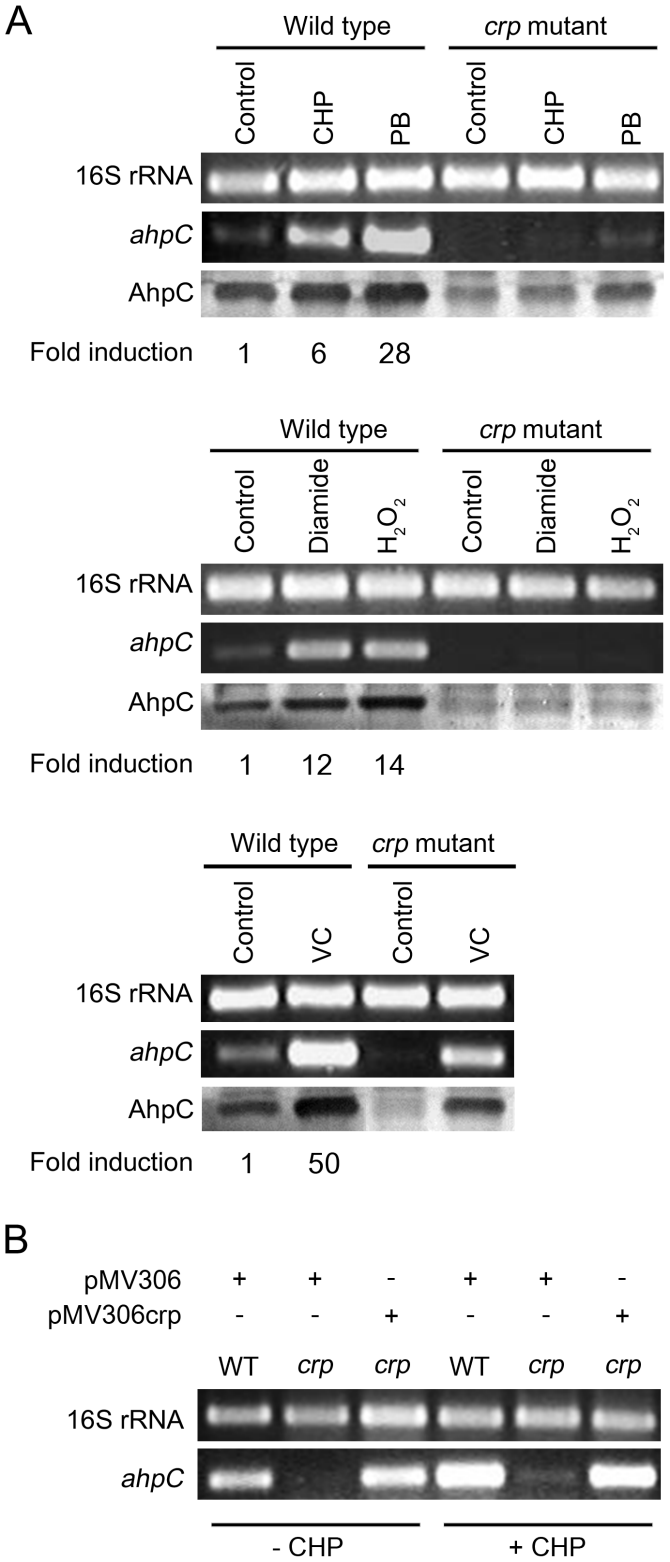
Expression of *ahpC* in the wild-type and *crp* mutant strains of *M. smegmatis* in response to various oxidative stresses and complementation of the *crp* mutant. (A) Transcript levels of *ahpC* were determined by RT-PCR and qRT-PCR. RT-PCR for 16S ribosomal RNA was performed to ensure that the same amounts of total RNA were employed for RT-PCR. Fold induction of *ahpC* expression determined by qRT-PCR indicates levels of *ahpC* mRNA in the strains treated with the oxidative-stress inducers relative to those in the untreated strains (control). Protein levels of AhpC were detected by means of Western blotting with polyclonal AhpC antibodies, and the results are presented below the RT-PCR results. Abbreviations: CHP, cumene hydroperoxide; PB, plumbagin; VC, sodium ascorbate. (B) The *crp* mutant (*crp*) was complemented by introducing pMV306crp. The wild-type (WT) and *crp* mutant strains harboring the pMV306 empty vector were used as controls. RT-PCR was performed using total RNAs isolated from the strains treated with CHP (+CHP) and untreated strains (−CHP).

Expression levels of *ahpC* in the *crp* mutant were compared with those in the wild-type strain by means of RT-PCR, qRT-PCR, and Western blotting after both strains were subject to oxidative stress generated by CHP (organic peroxide), H_2_O_2_, plumbagin (PB: superoxide generator), diamide (thiol-specific oxidant), and sodium ascorbate (VC: intracellular oxidative stress inducer). As controls, the *crp* mutant and wild-type strains without treatment of the oxidative stress reagents were included in the experiment (Figure 2A). When the wild-type strain was subject to CHP, H_2_O_2_, and diamide treatment, transcript levels of *ahpC* were increased 6-, 14-, and 12-fold, respectively, when compared with those determined for the untreated control strain. Treatment of the wild-type strain with plumbagin and ascorbate led to 28- and 50-fold induction of *ahpC* expression, respectively. In contrast, induction of *ahpC* expression by CHP, H_2_O_2_, and diamide was almost abolished in the *crp* mutant. When the *crp* mutant was treated with the strong inducers, plumbagin and ascorbate, induction of *ahpC* expression was significantly reduced but still observed. Western blotting analysis using polyclonal antibodies against AhpC also confirmed induction of *ahpC* by oxidative stress and requirement of Crp for the optimal expression of *ahpC*, although the extent of *ahp*C induction detected by Western blotting did not quantitatively well correlate with that determined by RT-PCR and qRT-PCR. The discrepancy in the induction fold of *ahpC* expression at transcriptional and translational levels might be due to posttranscriptional regulation or oxidative damages of translational machinery in the presence of the oxidative stress inducers. Expression of *ahpC* in the *crp* mutant in the presence and absence of CHP was restored by the introduction of the intact *crp* (*MSMEG_6189*) gene into the *crp* mutant (Figure 2B), indicating that a defect in *ahpC* expression observed for the *crp* mutant resulted from the inactivation of the *crp* (*MSMEG_6189*) gene.

**Figure 3:**
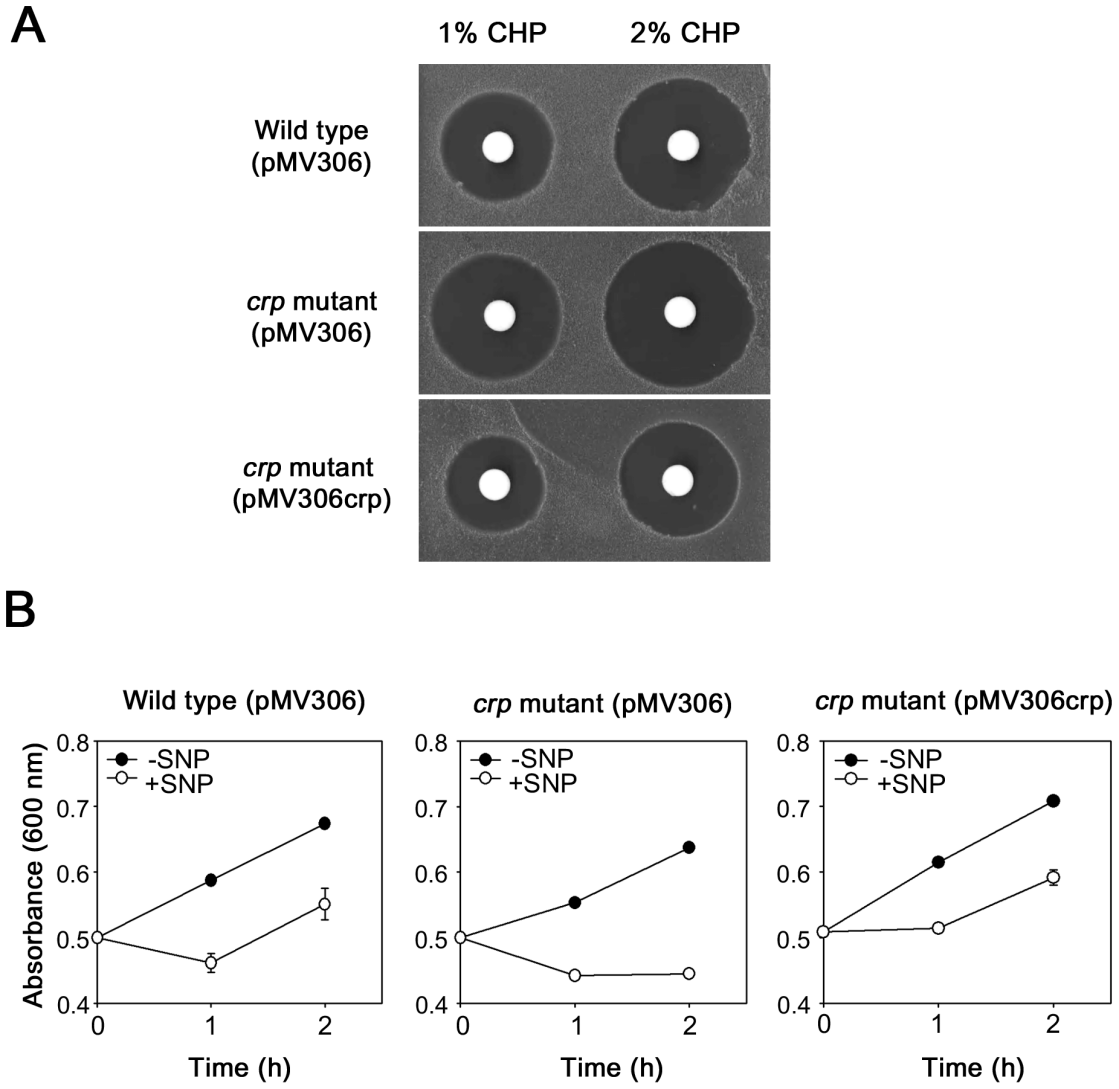
Susceptibility of the wild-type and *crp* mutant strains of *M. smegmatis* to CHP and NO. (A) Zone inhibition assay. The wild-type and *crp* mutant strains harboring pMV306 as well as the *crp* mutant complemented with pMV306crp were used. (B) Effect of NO treatment on growth of the wild-type and *crp* mutant strains of *M. smegmatis*. When the strains were grown to an OD_600_ of 0.5, SNP was added to the cultures with a final concentration of 10 mM and the cultures were further grown under the illumination of light (+SNP). As controls the strains without SNP treatment were included in the experiment (−SNP). The absorbance of the cultures was measured at 600 nm at intervals of 1 h. Growth of the cultures was monitored for only 2 h due to instability of SNP. The error bars indicate the deviations from the averages of two independent measurements.

AhpC is known to have the catalytic activity that reduces organic peroxides and peroxynitrite, thereby detoxifying them [2], [3], [4], [5]. Peroxynitrite is a reactive nitrogen intermediate (RNI) produced from the reaction of nitric oxide (NO) with superoxide that is a byproduct of aerobic metabolism [44]. To investigate whether the disruption of *crp* in *M. smegmatis* affects its susceptibility to CHP and NO, zone inhibition assay with CHP and growth inhibition assay with SNP (NO generator) were performed. As shown in Figure 3A, the *crp* mutant with the empty vector pMV306 gave rise to larger clear zones around the discs where 1% and 2% of CHP were applied than the wild type containing pMV306. The *crp* mutant complemented with pMV306crp resulted in even smaller growth-inhibitory zones than the wild type with pMV306. The result indicates that the inactivation of *crp* renders *M. smegmatis* more susceptible to CHP. To examine NO susceptibility of the *crp* mutant, the *crp* mutant with pMV306 grown to an OD_600_ of 0.5 was treated with SNP and growth of SNP-treated (+SNP) and untreated control (−SNP) strains was compared for 2 h by measuring the optical density of the cultures (Figure 3B). As controls, the wild-type strain with pMV306 and the complemented *crp* mutant were included in the experiment. The addition of 10 mM of SNP to the cultures had a bactericidal effect on both the wild-type and *crp* mutant strains with pMV306 and a bacteriostatic effect on the complemented *crp* mutant during the first hour of NO exposure. While growth resumed for the wild type with pMV306 and the complemented *crp* mutant 1 h after SNP treatment, that of the *crp* mutant with pMV306 did not, indicating that the *crp* mutant is more sensitive to NO than the wild type and the complemented *crp* mutant.

**Figure 4:**
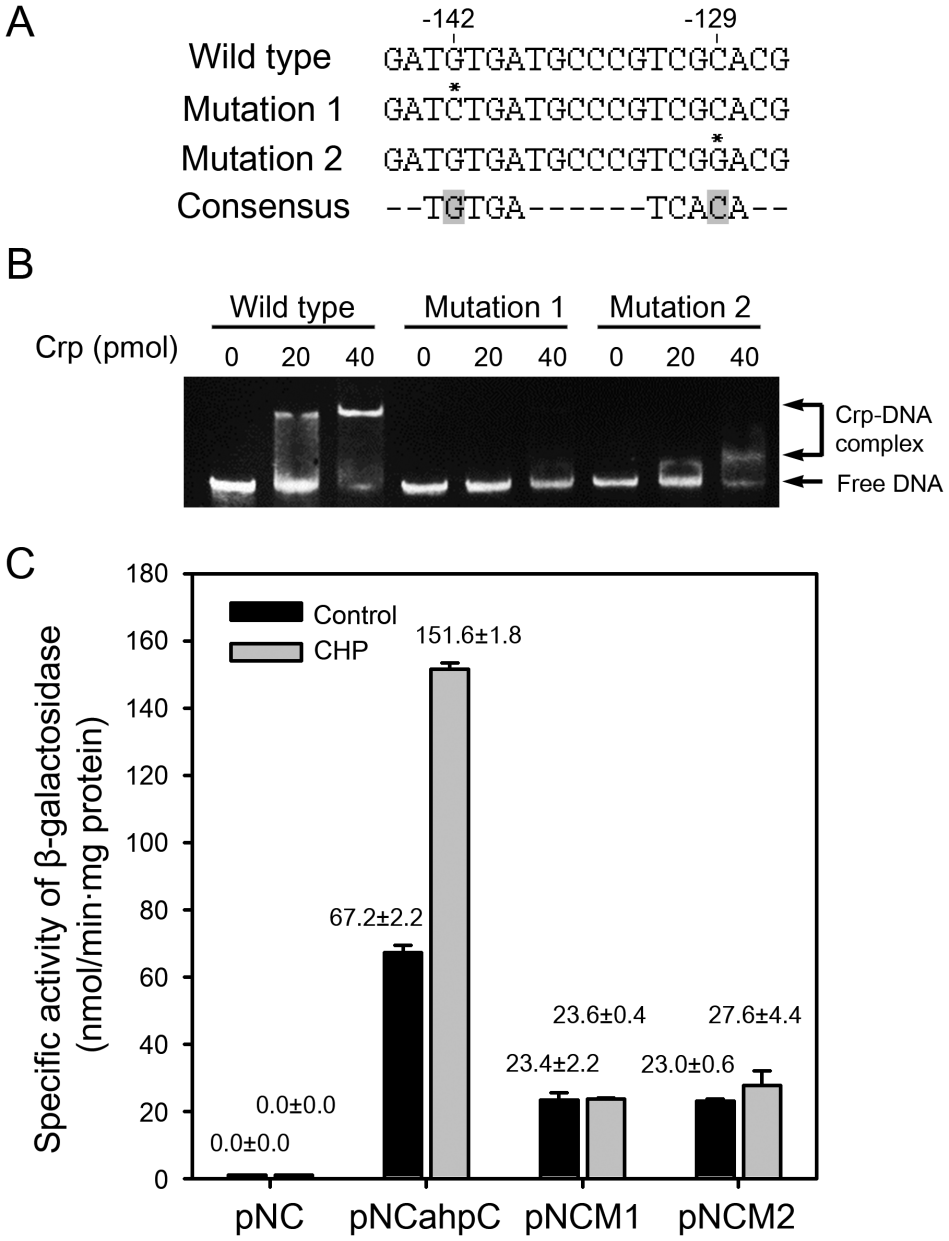
Effect of mutations in the IR1 sequence on Crp binding and *ahpC* expression. (A) Base substitution mutations of the conserved nucleotides within IR1. The consensus sequence of the Crp-binding sites is given at the bottom line. The mutated nucleotides are marked with the asterisks above the sequences. The numbers above the sequences indicate the positions of the mutated nucleotides relative to *ahpC*. (B) The 150-bp DNA fragments (9.3 ng, 100 fmol) containing the wild-type or mutated IR1 sequence (mutation 1 or 2) were incubated with various amounts of purified Crp in the presence of 100 µM cAMP. The amounts of Crp used are given above the lanes. The Crp-DNA reaction mixtures were subject to native PAGE. After electrophoresis, gels were stained with SYBR green EMSA gel staining solution. (C) Effect of the mutations within the IR1 sequence on the promoter activity of *ahpC.* The *ahpC* promoter activity was measured by determining β-galactosidase activity. *M. smegmatis* wild-type strains harboring pNCahpC, pNCM1, and pNCM2 were grown to an OD_600_ of 0.45 to 0.5 and treated with CHP or DMSO (the solvent for CHP stock solution: control). The cultures were further grown for 1 h. Cell-free crude extracts were used to measure β-galactosidase activity. All values are the means of two independent experiments. The error bars indicate the deviations from the means.

To assess the role of IR1 on *ahpC* expression, point mutations were introduced into the IR1 sequence by means of site-directed mutagenesis (Figure 4A). The shaded G and C nucleotides of the IR1 sequence corresponding to the strictly conserved nucleotides in the mycobacterial Crp-binding sites [45] were substituted with C and G, respectively. To ascertain whether the IR1 sequence is required for Crp binding *in vitro*, we performed EMSA with purified Crp (MSMEG_6189) of *M. smegmatis* and three types of 150-bp DNA fragments containing the wild-type or mutated IR1 sequences (mutation 1 and mutation 2) in the presence of 100 µM cAMP. When the wild-type DNA fragment was employed, the increasing amounts of Crp-DNA complexes were formed in proportion to the amounts of Crp protein. In contrast, the formation of Crp-DNA complexes was abolished when the DNA fragment containing mutation 1 (G to C mutation) was used in EMSA assay. The DNA fragment containing mutation 2 (C to G mutation) exhibited weak retarded bands that were smeared and closely migrated to free DNA bands, compared with the wild-type DNA fragment, indicative of weak interactions between Crp and the DNA fragment containing mutation 2 (Figure 4B).

We next examined the effect of IR1 mutations on *ahpC* expression using *ahpC*::*lacZ* transcriptional fusions. pNCahpC is a pNC-based *ahpC*::*lacZ* transcriptional fusion plasmid. pNCM1 and pNCM2 have the same constructs as pNCahpC except for mutation 1 and mutation 2 within IR1, respectively. The wild-type strains of *M. smegmatis* harboring pNC, pNCahpC, pNCM1, and pNCM2 were aerobically grown and treated with CHP. Promoter activities of *ahpC* were determined by β-galactosidase assay using cell-free crude extracts. As controls, the same strains without CHP treatment were included in the experiment. Here we chose CHP, the mildest inducer used in Figure 2A, as an inducer of *ahpC* expression, since treatment of cell crude extracts for 1 h with plumbagin, H_2_O_2_, and ascorbate led to a significant decrease in β-galactosidase activity (data not shown). The wild-type strain harboring the empty pNC vector showed virtually no β-galactosidase activity regardless of CHP treatment. In the case of the wild-type strain containing pNCahpC, 2.3-fold induction of *ahpC* expression by CHP treatment was observed relative to the control without CHP treatment. The CHP-untreated strains containing pNCM1 and pNCM2 showed basal levels of β-galactosidase activity that amounted to approximately 30% of those detected in the untreated strain with pNCahpC. Expression of *ahpC* was not induced in the wild-type strain with pNCM1 by CHP treatment and the strain with pNCM2 showed a marginal increase in *ahpC* expression by CHP treatment (Figure 4C). Taken together, the results obtained from both EMSA and promoter activity assay indicate that IR1 serves as an activator-binding site for Crp and that the conserved nucleotides G and C within the IR1 sequence are important for Crp binding and activation of *ahpC* expression.

### Cellular levels of cAMP affect *ahpC* expression

**Figure 5:**
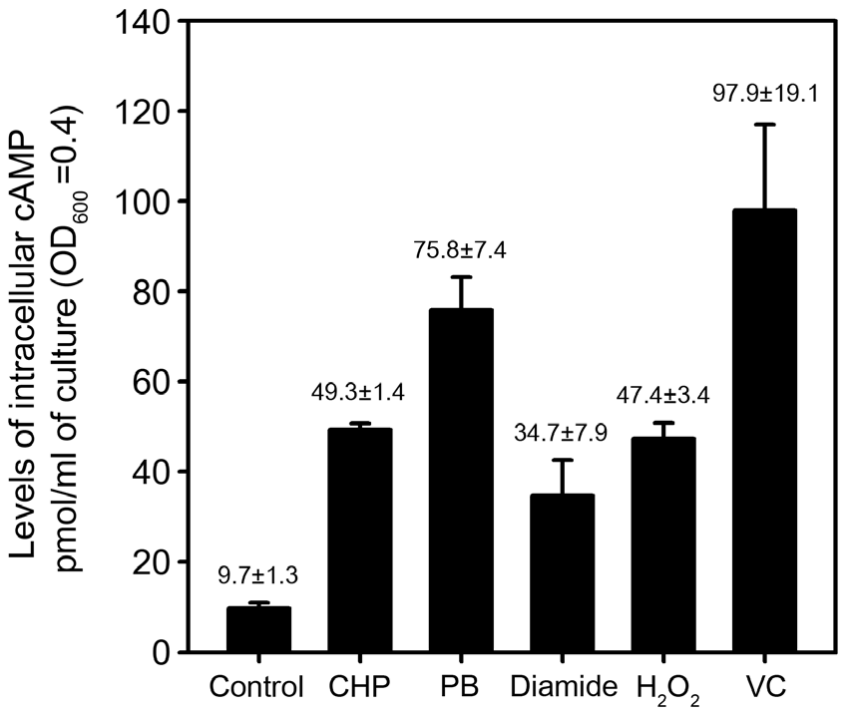
Determination of intracellular cAMP levels. The wild-type strain of *M. smegmatis* was grown to an OD_600_ of 0.45 to 0.5 and treated with various oxidative stress inducers. The untreated wild-type strain was included in the experiment as a control. Levels of intracellular cAMP were determined by using the DetectX Direct Cyclic AMP Enzyme Immunoassay kit. All values are the means of two independent experiments. The error bars indicate the deviations from the means.

**Figure 6:**
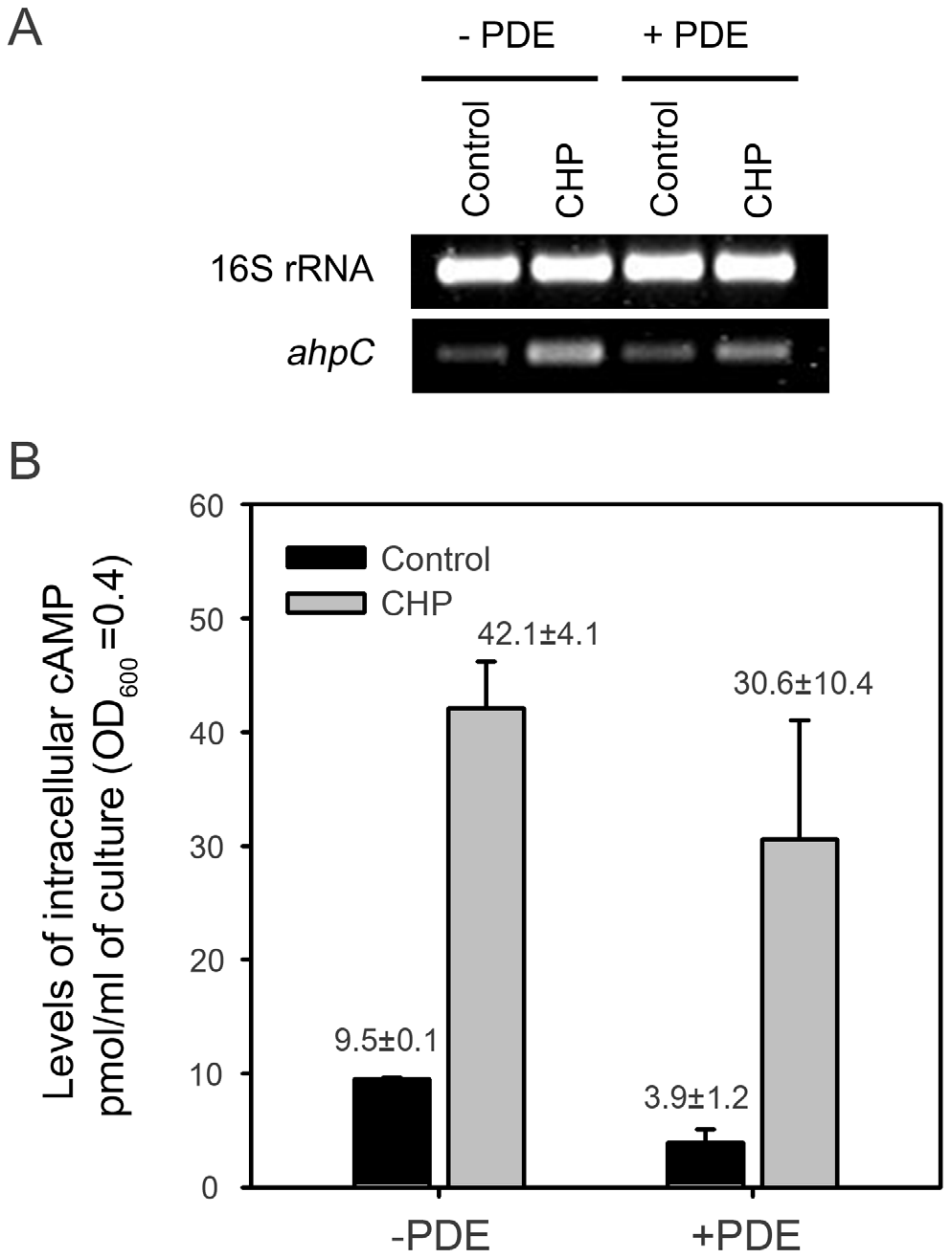
Effect of cAMP phosphodiesterase (PDE) overexpression on *ahpC* expression in *M. smegmatis*. (A) Expression levels of *ahpC* were determined by means of RT-PCR. For induction of *rv0805* encoding PDE of *M. tuberculosis*, the wild-type strain of *M. smegmatis* with pMHpdeHis (+PDE) was grown to an OD_600_ of 0.45 to 0.5 in the presence of 0.2% acetamide. The cultures were treated with CHP or DMSO (control) and further grown for 1 h to induce *ahpC* expression. The wild-type strain harboring pMH201 (−PDE) was included in the experiment. (B) Intracellular cAMP levels in the strains described in the panel A were determined. All values are the means of three independent experiments. The error bars indicate the standard deviations.

As shown in Figure 5, intracellular levels of cAMP were determined for the wild-type strain of *M. smegmatis* treated with CHP, PB, diamide, H_2_O_2_, and ascorbate. The untreated wild-type strain was included as a control. When the strain was treated with CHP, PB, diamide, H_2_O_2_, and ascorbate, intracellular levels of cAMP were increased 5.1-, 7.8-, 3.6-, 4.9- and 10.1-fold, respectively. This result suggests the possibility that increased levels of cAMP under oxidative stress conditions might contribute to enhancement of *ahpC* expression via Crp. We next examined whether expression of *ahpC* in *M. smegmatis* was affected by changes in the cellular level of cAMP. For this experiment, we employed two *M. smegmatis* strains: one is the wild-type strain containing pMHpdeHis where the cyclic nucleotide phosphodiesterase (PDE, Rv0805) gene of *M. tuberculosis* is under the control of an acetamide-inducible promoter and the other is the wild-type strain containing pMH201, the empty vector of pMHpdeHis. Both strains were grown aerobically to an OD_600_ of 0.45 to 0.5 in the presence of 0.2% acetamide and further grown for 1 h either with or without CHP treatment. Under both CHP-treated and untreated conditions a decrease in intracellular cAMP levels was observed in the strains carrying pMHpdeHis, when compared with the strains carrying pMH201 (Figure 6), indicating that the expressed PDE of *M. tuberculosis* can hydrolyze cAMP in *M. smegmatis* cells. The determination of *ahpC* expression by means of RT-PCR revealed that *ahpC* expression was significantly reduced in the CHP-treated strain with pMHpdeHis relative to the CHP-treated strain with pMH201. We performed this experiment three times independently and the results were reproducible. These results strongly indicate that cellular levels of cAMP are reflected to control *ahpC* expression in *M. smegmatis*.

### Role of IR2 as a *cis*-regulatory element in the regulation of *ahpC* expression

**Figure 7:**
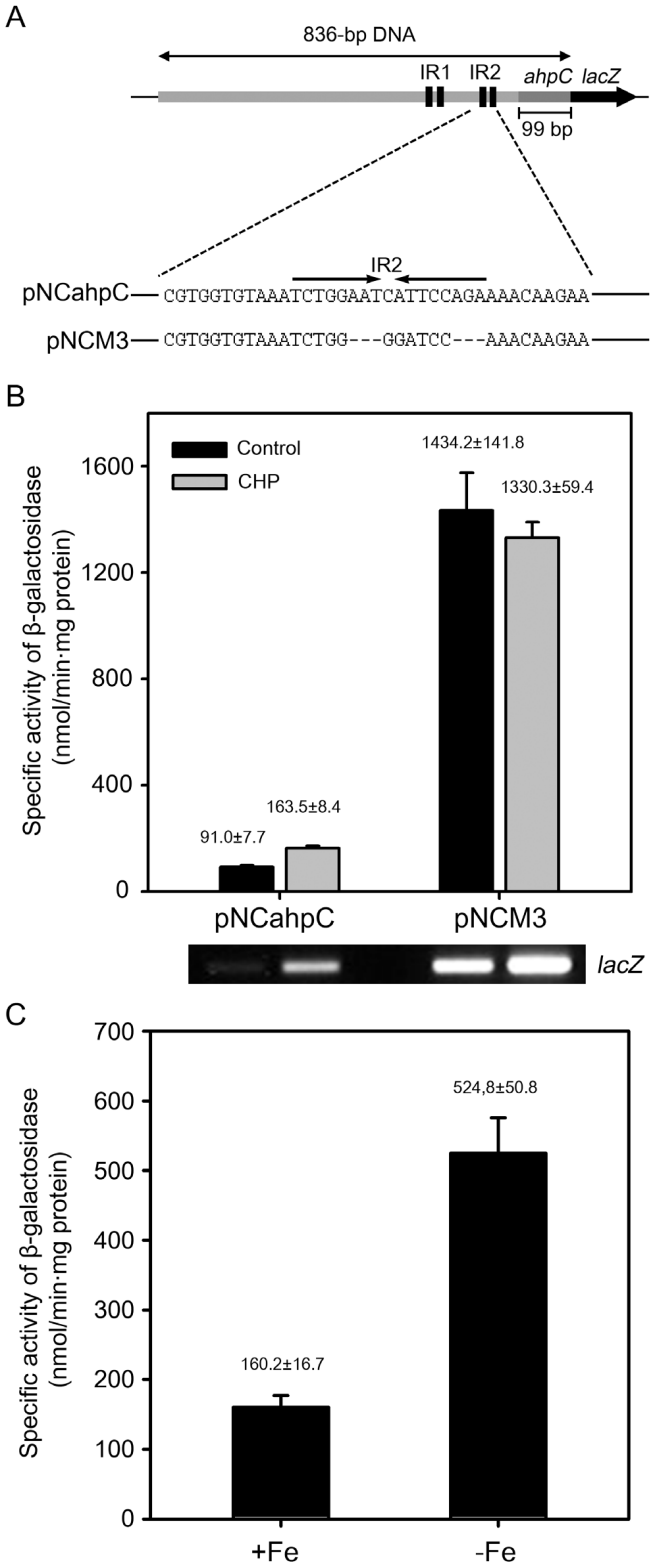
Effect of deletion of the IR2 sequence on *ahpC* expression and derepression of *ahpC* expression under iron-depleting conditions. (A) Schematic diagram of pNCM3. The *lacZ* transcriptional fusion plasmid pNCM3 carries the same DNA fragment as pNCahpC except for the substitution of a part of IR2 with the BamHI recognition sequence. (B) *M. smegmatis* wild-type strains harboring pNCahpC and pNCM3 were grown to an OD_600_ of 0.45 to 0.5, and treated with CHP or DMSO (control). The cultures were further grown for 1 h. Cell-free crude extracts were used to measure β-galactosidase activity. Expression levels of *lacZ* in the wild-type strains carrying pNCahpC and pNCM3 were also determined by RT-PCR and the result is presented below the graph. All values are the means of two independent experiments. The error bars indicate the deviations from the means. (C) The wild-type strain of *M. smegmatis* harboring pNCahpC was grown in 7H9 medium to an OD_600_ of 1.5 to 2.0. Pre-cultured cells were washed twice with the original volume of MOPS medium supplemented with 0.02% Tween 80 and resuspended to the same volume of MOPS medium. 1 ml of the preculture was inoculated to 100 ml of MOPS medium supplemented with either 50 µM FeCl_3_ (+Fe) or 100 µM 2,2′-Dipyridyl (iron chelator) (−Fe). The strain was grown to an OD_600_ of 0.45 to 0.5 and harvested. Expression levels of *ahpC* were determined by performing β-galactosidase assay. The error bars indicate the deviations from the means of the two independent experiments.

pNCM3 is a pNCahpC derivative carrying the same DNA fragment as pNCahpC except for the replacement of a 12-bp portion of IR2 with the BamHI recognition sequence (Figure 7A). Using pNCM3, the role of IR2 in the regulation of *ahpC* expression was examined. The wild-type strains harboring pNCahpC and pNCM3 were grown either with or without CHP treatment and promoter activities of *ahpC* in the strains were determined by means of β-galactosidase assay. Expression of *ahpC* was induced by CHP treatment in the wild-type strain with pNCahpC (Figure 7B). Expression of *ahpC* was strongly derepressed in the strain with pNCM3 in the presence and absence of CHP treatment. A slight decrease in *ahpC* expression by CHP was observed in the strain with pNCM3. When *ahpC* expression was measured by RT-PCR using primers for the detection of *lacZ*, transcription of *lacZ* on pNCM3 was shown to be slightly induced in the CHP-treated wild-type strain carrying pNCM3 relative to the untreated strain with pNCM3, indicating that the expression level of *ahpC* determined by β-galactosidase assay was underestimated in the presence of CHP, when compared with that determined by RT-PCR. The result indicates that IR2 serves as a repressor-binding site (operator) for the regulation of *ahpC* expression.

**Figure 8:**
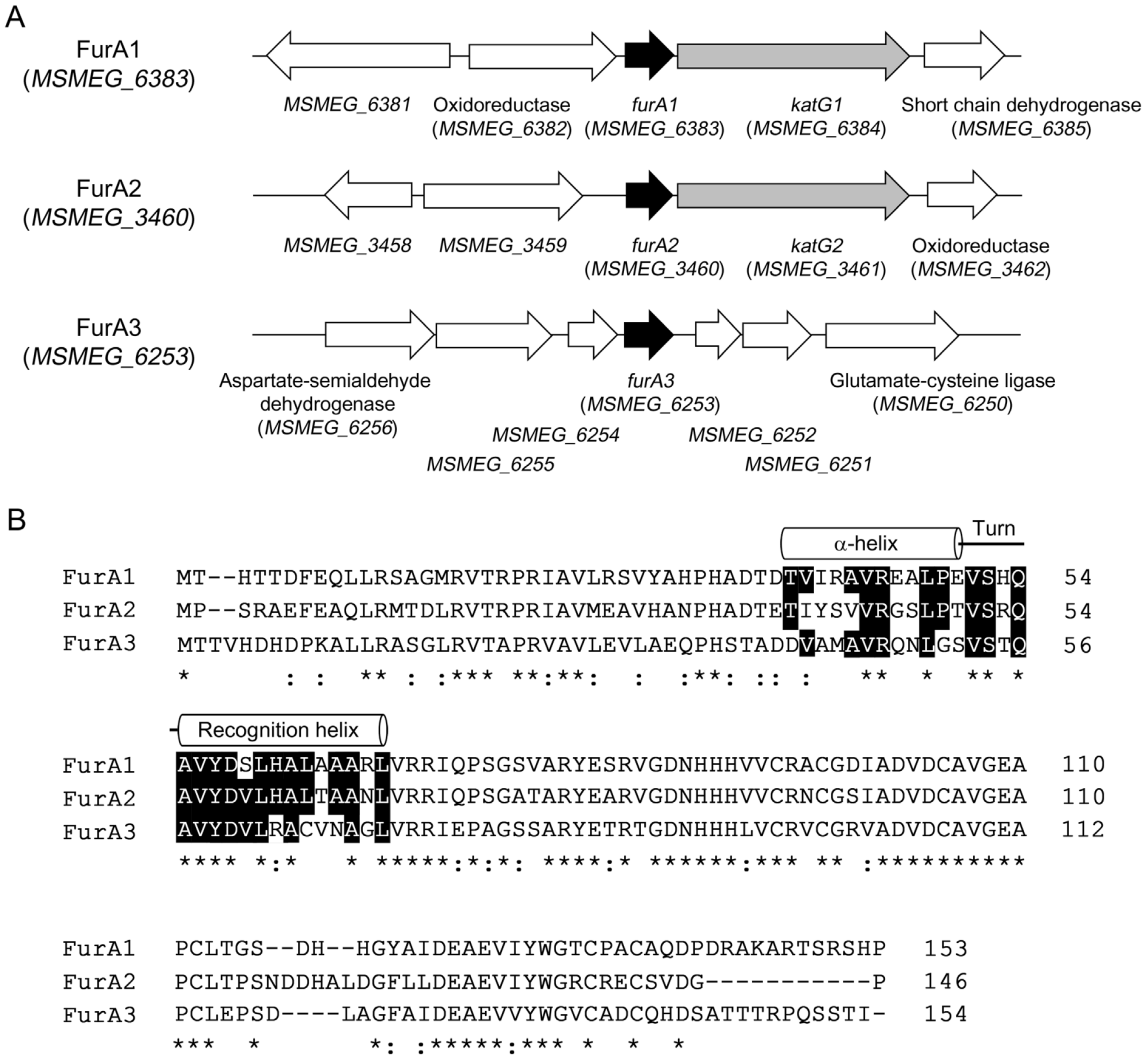
Genetic organization of the *furA1*, *furA2*, and *furA3* loci in *M. smegmatis* mc^2^155 (A) and multiple alignment of the FurA homologs (B). Multiple alignment was generated by using Clustal W. The position of the helix-turn-helix motif was extrapolated from the determined three-dimensional structure of FurA [58] and indicated with two cylinders. Identical and conservatively substituted residues are indicated by asterisks and colons, respectively. The amino acid residues, which comprise the helix-turn-helix motif and are conserved in two and three FurA homologs, are highlighted in black.

The high similarity of the IR2 sequence to the known FurA-binding consensus sequence led us to speculate that FurA is involved in repression of *ahpC*. We identified three genes encoding FurA homologs (MSMEG_6383, MSMEG_3460, and MSMEG_6253) from *M. smegmatis* genome (Figure 8A). The genes encoding MSMEG_6383 (FurA1) and MSMEG_3460 (FurA2) are located immediately upstream of the duplicated *katG* genes coding for peroxidase-catalases, MSMEG_6384 (KatG1) and MSMEG_3461 (KatG2), respectively. All the amino acid residues involved in the coordination of the structural Zn^2+^ and regulatory Fe^2+^ ions are conserved in FurA1, FurA2, and FurA3, and their DNA-binding helix-turn-helix domains (amino acids 36 to 68 for FurA1) are relatively well conserved (Figure 8B). Phylogenetic analysis using their entire amino acid sequences revealed that FurA1 is more closely related with FurA2 than with FurA3 (data not shown). On account of difficulties in the construction of a *furA* triple mutant, we did not directly examine the involvement of FurA in *ahpC* expression by determining *ahpC* expression in the *furA* triple mutant. Instead, we determined the effect of iron depletion on *ahpC* expression on the basis of the fact that FurA is a Fe^2+^-dependent regulatory protein [46]. As shown in Figure 7C, the expression level of *ahpC* was increased 3.3-fold in *M. smegmatis* grown under iron-depleting conditions, compared with the control *M. smegmatis* strain grown in the medium replete with iron. This result indicates the involvement of an iron-dependent regulator in *ahpC* repression, possibly the FurA protein.

## Discussion

### Crp is an activator for *ahpC* expression in *M. smegmatis*

Two genes encoding Crp homologs (MSMEG_6189 and MSMEG_0539) occur in *M. smegmatis* genome. The primary structure of MSMEG_6189 is almost identical to Crp_Mtb_ (98% sequence identity), while MSMEG_0539 possesses 78% identity to Crp_Mtb_. Expression of *ahpC* in the *crp* (*MSMEG_6189*) mutant strain of *M. smegmatis* was shown to be almost abolished or significantly decreased under induction conditions of *ahpC*, compared with the wild-type strain subject to the same conditions. Furthermore, purified Crp (MSMEG_6189) was shown to specifically bind to the IR1 sequence which has a high similarity to the Crp-binding consensus sequence. Both results indicate that Crp bound to IR1 serves as an activator for *ahpC* expression and that MSMEG_6189 is a major functional Crp protein in *M. smegmatis*. In many cases the role of Crp as an activator or a repressor is dependent on the position of its binding site relative to the transcription start point of its target gene. The Crp-binding sites located upstream of the promoters usually serve as activation sites and the binding sites located adjacent to the transcription start point serve as repression sites [47], [48]. In good agreement with this, the IR1 sequence is centered at −81.5 relative to the transcriptional start point of *ahpC* and serves as an activator-binding site. Since AhpC can remove organic peroxides, H_2_O_2_, and peroxynitrite (ONOO^−^) by using its peroxidase activity catalyzing the reduction of the peroxide linkage (-O-O-), increased susceptibility of the *crp* mutant strain to CHP and NO relative to the wild-type strain is attributable to a defect in *ahpC* expression in the *crp* mutant. AhpC was suggested to protect mycobacteria from deleterious effects of organic peroxides and peroxynitrite [5], [16], [49]. The production of reactive oxygen intermediates (ROIs) and RNIs by macrophages is considered to be the major mechanism restraining *M. tuberculosis* proliferation *in vivo* [50], [51]. The fact that a *crp* mutant strain of *M. tuberculosis* showed a reduced survival rate within macrophages and attenuated virulence in a murine infection model implies that Crp is also involved in the induction of genes related to adaptation to and defense mechanisms against ROIs and RNIs in *M. tuberculosis* [18]. There is a sequence (GGTGT-N_6_-TCACC) with a partial similarity to the Crp-binding consensus sequence (TGTGA-N_6_-TCACA), which is located 77 bp upstream of the *M. tuberculosis ahpC* gene [18]. Microarray analysis revealed that the *ahpC* gene is downregulated in a *crp* mutant of *M. tuberculosis* [18], indicating that *ahpC* of *M. tuberculosis* is also positively regulated by Crp.

### The IR2 sequence is a *cis*-acting regulatory element involved in the negative regulation of *ahpC* expression

The location of a *cis*-regulatory DNA sequence determines its function in many cases. Transcriptional repressors bind predominantly to positions either overlapping with or downstream of the promoters of their target genes to prevent RNA polymerase from closed or open complex formation. The IR2 site is located between the promoter and the start codon of *ahpC*. Deletion of IR2 made *ahpC* strongly derepressed regardless of the presence and absence of CHP and its sequence (TCTGGAAT-N-ATTCCAGA) is very similar to the FurA-binding sites (TCTTGACT-N-ATTCCAGA: the nucleotides that are different from those of IR2 are underlined) located upstream of the autoregulated *M. tuberculosis furA* and *M. smegmatis furA1* genes [43], [52]. Although we did not put forward direct evidence to support the involvement of FurA in *ahpC* expression due to the presence of three FurA homologs in *M. smegmatis*, derepression of *ahpC* expression under iron-depleting conditions together with the resemblance of the IR2 sequence to FurA-binding sequences strongly indicate that *ahpC* is under the negative regulation of FurA. Further study using a *furA* triple mutant is required to prove this suggestion and the construction of the mutant is under way. It was previously demonstrated by means of immunoblot analysis that the steady-state level of AhpC was not affected by inactivation of the *furA1* gene in *M. smegmatis* [53], which can be explained by the presence of multiple FurA homologs. The functionality of FurA was suggested to be controlled by NO and ROIs [43], [54], [55]. NO and ROIs can inactivate the Fe^2+^-containing FurA protein, thereby inducing the genes that are under the negative regulation of FurA. This property of FurA enables it to serve as an RNI- and ROI-responsive regulator in addition to an iron-responsive regulator.

### Cellular levels of cAMP correlate with the expression level of *ahpC*

The finding that more than 40% of genes in the Crp regulon overlap with hypoxia- and starvation-stimulated genes in *M. tuberculosis* gives a clue that Crp might activate expression of Crp regulon in response to increased levels of cAMP under hypoxic and starvation conditions [45], [56], [57]. Recently it was demonstrated in *M. tuberculosis* that increased cellular levels of cAMP by heat stress or exogenous dibutyryl cAMP treatment led to upregulation of some heat stress-induced genes that have the Crp-binding sequences in their control regions, strongly indicating that the cellular cAMP level correlates with expression levels of the Crp regulon [66]. In this study, we observed both reduced expression of *ahpC* in a PDE-overexpressed strain of *M. smegmatis* relative to the control strain and an increase in cellular levels of cAMP under oxidative stress conditions. *lacZ* expression from pNCM3 without IR2 was shown to be slightly induced by CHP treatment (see Figure 7B RT-PCR), which appears to be the consequence of elevated cAMP levels by CHP treatment. Taken together, these results strongly suggest that induction of *ahpC* expression under oxidative stress conditions probably results from a combinatory effect of both inactivation of FurA by oxidative stress and activation of Crp in response to an increase in cellular levels of cAMP.
